# Relationship between cardio-ankle vascular index value and stroke in hypertension patients cardio-ankle vascular index ≧9

**DOI:** 10.1371/journal.pone.0321298

**Published:** 2025-04-24

**Authors:** Jinbo Liu, Shantong Jiang, Xuechen Cui, Xiu Bai, Huan Wen, Hongwei Zhao, Hongyu Wang

**Affiliations:** Department of Vascular Medicine, Peking University Shougang Hospital, Beijing, China; University of Milan, ITALY

## Abstract

**Backgrounds:**

The cardio-ankle vascular index (CAVI) is a new index of arteriosclerosis. The present study investigated the relationship between CAVI value and stroke in hypertension patients, especially the prevalence of stroke in patients with CAVI ≧9.

**Methods:**

735 patients (M/F 293/442) with or without hypertension from Department of Vascular Medicine from 01/01/2012–31/21/2014 were divided into four groups: group 1: non-hypertension patients with CAVI<9, group 2: non-hypertension patients with CAVI ≧9, group 3: hypertension patients with CAVI<9, group 4: hypertension patients with CAVI ≧9. CAVI was measured by VS-1000 apparatus.

**Results:**

Prevalence of stroke and coronary artery disease were significantly higher in group 2 than in group 1. And the prevalence of stroke and coronary artery disease were also significantly higher in group 4 than in group 3. In addition, the level of right intima-media thickness (RIMT) was significantly higher in group 4 than in group 3 (0.102±0.025 vs 0.094±0.023, p<0.05). Multiple linear regressions showed that CAVI and age were independent associating factors of stroke in all patients (β=0.268, p=0.040; β=0.135, p<0.001; respectively). CAVI was an independent associating factors of stroke in hypertension patients (β=0.398, p<0.001).

**Conclusion:**

The prevalence of stroke was higher in hypertension patients with CAVI ≧9 than in hypertension patients with CAVI<9, with higher level of right intima-media thickness. CAVI was an independent associating factors of stroke in hypertension patients.

## Introduction

Hypertension is one of the most common vascular diseases in the world. In rural China, the incidence rate of hypertension remains high, accompanied by low awareness, treatment and control rates [1]. Hypertension is also the main cause of stroke. Stroke represents a major challenge for the health system, which could cause disability and increase the burden of family and society [2]. Arteriosclerosis is the pathophysiological change during the development of stroke. Arteriosclerosis could be evaluated by arterial stiffness, which is an important predictor of cardiovascular events and all-cause mortality [3]. Previous studies showed that cardio-ankle vascular index (CAVI) is a reliable indicator for evaluating arterial stiffness [4].

CAVI was reportedly positively correlated with homocysteine and tended to increase in patients with hypertension [5,6]. Recent study showed that CAVI was positively associated with high glycaemia and high blood pressure components, and age might have a more pronounced effect on CAVI [7]. Shirai K showed that CAVI was higher in persons with diabetes mellitus, metabolic syndrome, hypertension and smoking [8], and patients with higher CAVI exhibited a worse cognitive function. CAVI could assess early manifestations of dementia [9]. Kubota reported that with the increase of CAVI levels, the incidence of heart disease and cerebrovascular disease increased in 3 years [10].

However, there was little research about the relationship between CAVI value and stroke in hypertension patients. Arteriosclerosis caused by hypertension is an important cause of stroke Therefore, evaluating the relationship between the degree of arteriosclerosis and stroke in hypertensive patients is of great significance for the prevention and treatment of cerebrovascular diseases, especially exploring some non-invasive and simple evaluation technical parameters, such as the CAVI. So in the present study, we investigated the the relationship between CAVI value and stroke in hypertension patients, especially the prevalence of stroke in patients with CAVI≧9.

## Materials and methods

### Patients

This was a retrospective study that included patients who visited the Department of Vascular Medicine at Peking University Shougang Hospital from 01/01/2012–31/21/2014. According to our department’s requirements, all patients were asked if we could use their clinical data for scientific research in the future. If the patients agreed, they would sign an informed consent form. The exclusion criteria for this study were: patients with diabetes mellitus, heart failure, renal function impairment, liver function impairment, systemic inflammatory diseases, infectious disease or cancer were excluded. Starting from January 1, 2022, with the support of the fund project and ethical support, we began to access the data retrospectively. Finally, 735 patients (M/F 293/442) with or without hypertension from 01/01/2012–31/21/2014 were enrolled into our study. Hypertension was defined as blood pressure measurement ≧140/90mmHg in three occasions at rest or patients with known cases of diagnosed hypertension before and taking antihypertensive drugs at present. Coronary artery disease was defined as the narrowing or blockage of coronary artery diagnosed by angiography. Stroke was diagnosed by magnetic resonance spectrum. All participants gave their written informed consent. This study was approved by the ethics committee of Peking University Shougang Hospital (reference number, SGYYZ202105).

### The assessment of CAVI

CAVI was recorded using a VaseraVS-1000 vascular screening system (Fukuda Denshi, Tokyo, Japan), with the patients resting in a supine position. The patients were placed flat on the examination bed. ECG electrodes were placed on both wrists; a microphone to detect heart sounds was placed on the sternum, and cuffs were wrapped around both arms and ankles. After automatic measurements, the data obtained were analyzed using the software, and the CAVI value was automatically obtained.

### Laboratory measurements

Blood samples were drawn from an antecubital vein in the morning after overnight fasting and collected into vacuum tubes containing EDTA for the measurement of plasma lipid and lipoprotein levels. Fasting plasma glucose (FPG), Total cholesterol (TC), HDL-C, and TG levels were analyzed by colorimetric enzymatic assays with the use of an autoanalyzer (HITACHI-7170, Hitachi, Tokyo, Japan) at the central chemistry laboratory of the Peking University Shougang Hospital. LDL-C levels were calculated.

### Statistical analysis

SPSS (version 26.0) was used for the statistical analysis.The differences between groups were analyzed by *t*-test. Proportions were analyzed by χ^2^ -test. Correlation coefficient was done to find linear relation between different variables using Pearman correlation analysis. Multiple linear regressions were used to estimate the coefficients of the linear equation, involving independent variables that affected the value of the dependent variables. Values were shown as mean ± SD unless stand otherwise. *p* < 0.05 (2-tailed) was considered statistically significant.

## Results

### Clinical characteristics of the study participants

The clinical characteristics of study participants were shown in Table 1. Our results showed that the levels of age, CAVI, systolic blood pressure (SBP), diastolic blood pressure(DBP), pulse pressure, uric acid and homocysteine were significantly higher in hypertension patients than in non-hypertension patients. The prevalence of stroke and coronary artery disease were higher in hypertension patients. And the drugs usage such as statins, angiotensin converting anzyme inhibitior/angiotensin Ⅱ receptor antagonist (ACEI/ARB), calcium channel blocker (CCB), beta blockers, nitrates and anti-platelet drugs were more in hypertension patients than in non-hypertension patients.

**Table 1:** Clinical characteristics in the entire study group.

Characterisitics	Non-hypertension patients N=293	Hypertension patients N=442	P value
Age (year)	58.86 ± 11.14	5.59 ± 12.18	<0.001
Male/Female	122/171	213/229	0.084
Coronary artery diseas(No. %)	65, 22%	211, 48%	<0.001
Smoking (No. %)	78, 27%	144, 33%	0.076
Stroke (No. %)	46, 16%	147, 33%	<0.001
Statins (No. %)	74, 25%	164, 37%	0.001
ACEI/ARB (No.%)	6, 2%	166, 38%	<0.001
CCB (No.%)	5, 2%	222, 50%	<0.001
Beta blockers (No.%)	27,9%	110, 25%	<0.001
Nitrates (No.%)	21, 7%	72, 16%	<0.001
Anti-platelet drug	67, 23%	174, 39%	<0.001
BMI (Kg/M^2)	24.42 ± 3.58	25.58 ± 3.34	<0.001
CAVI	7.95 ± 1.34	8.49 ± 1.53	<0.001
SBP (mmHg)	129.35 ± 16.22	144.77 ± 19.43	<0.001
DBP (mmHg)	79.71 ± 9.86	86.08 ± 11.30	<0.001
Pulse pressure (mmHg)	49.62 ± 11.54	58.72 ± 15.78	<0.001
Creatinine (umol/L)	64.54 ± 16.06	71.02 ± 25.82	<0.001
FPG (mmol/L)	5.46 ± 0.92	5.51 ± 0.93	0.536
UA(umol/L)	297.86 ± 75.51	326.80 ± 82.83	<0.001
TC (mmol/L)	5.07 ± 1.09	4.66 ± 1.14	<0.001
HDL-C (mmol/L)	1.30 ± 0.34	1.20 ± 0.29	<0.001
LDL-C (mmol/L)	3.15 ± 0.91	2.82 ± 0.91	<0.001
TG (mmol/L)	1.72 ± 1.35	1.64 ± 1.13	0.367
Homocysteine (umol/L)	12.78 ± 6.80	15.76 ± 8.20	<0.001
RIMT (cm)	0.093 ± 0.025	0.096 ± 0.024	0.190
LIMT (cm)	0.098 ± 0.033	0.110 ± 0.072	0.064

Note: ACEI: angiotensin converting anzyme inhibitior; ARB: angiotensin Ⅱ receptor antagonist; CCB: calcium channel blocker; BMI: body mass index; CAVI: cardio-ankle vascular index; SBP: systolic blood pressure; DBP: diastolic blood pressure; FPG: fasting plasma glucose; UA: uric acid; TC: cholesterol; LDL-C: low-density lipoprotein cholesterol; HDL-C: high-density lipoprotein cholesterol; TG: triglycerides; RIMT: right intima-media thickness; LIMT:left intima-media thickness.

Next, all patients were divided into four groups: group 1: non-hypertension patients with CAVI<9, group 2: non-hypertension patients with CAVI ≧9, group 3: hypertension patients with CAVI<9, group 4: hypertension patients with CAVI ≧9. As shown in Table 2, the prevalence of stroke and coronary artery disease were significantly higher in group 2 than in group 1. And the prevalence of stroke and coronary artery disease were also significantly higher in group 4 than in group 3. In addition, the level of right intima-media thickness (RIMT) was significantly higher in group 4 than in group 3 (0.102±0.025 vs 0.094±0.023, p<0.05). However, there were no significant difference about the composition of gender, smoking, drugs usage such as statins, ACEI/ARB, CCB, beta blockers, nitrates and anti-platelet drugs between group 3 and goup 4.

**Table 2:** Clinical characteristics in different groups in non-hypertension patients.

Characterisitics	Group 1 N=222	Group 2 N=71	Group 3 N=287	Group 4 N=155	P value
Age (year)	55.26 ± 9.28	70.14 ± 8.70*	61.81 ± 11.74*^,^^#^	72.95 ± 9.40*^,^^$^	<0.001
Male/Female	86/136	36/35	131/156	82/73	0.040
Coronary artery diseas(No. %)	38, 17%	27, 38%*	123, 43%	88, 57%^$^	<0.001
Smoking (No. %)	56, 25%	22, 31%	94, 33%	50, 32%	0.217
Stroke (No. %)	24, 11%	22, 31%*	72, 25%	74, 48%^$^	<0.001
Statins (No. %)	52, 23%	22, 31%	100, 35%	64, 41%	0.002
ACEI/ARB (No.%)	4, 2%	2, 3%	105, 37%	61, 39%	<0.001
CCB (No.%)	5, 2%	0, 0%	137, 48%	85, 55%	<0.001
Beta blockers (No.%)	23,10%	4, 6%	65, 23%	45, 29%	<0.001
Nitrates (No.%)	14, 6%	7, 10%	35, 12%	37, 24%$	<0.001
Anti-platelet drug	51, 23%	16, 23%	114, 40%	60, 39%	<0.001
BMI (Kg/M^2)	24.97 ± 3.50	22.69 ± 3.32*	26.18 ± 3.18*^,^^#^	24.50 ± 3.36^#^^,^^$^	<0.001
CAVI	7.39 ± 0.98	9.76 ± 0.83*	7.67 ± 1.06*^,^^#^	10.00 ± 1.04*^,^^$^	<0.001
SBP (mmHg)	127.30 ± 14.65	135.84 ± 19.11*	141.73 ± 18.54*	150.84 ± 19.65*^,^^#^^,^^$^	<0.001
DBP (mmHg)	80.01 ± 9.82	78.76 ± 10.00	86.00 ± 10.87*^,^^#^	86.34 ± 12.02*^,^^#^	<0.001
Pulse pressure (mmHg)	47.26 ± 9.36	57.09 ± 14.37*	55.77 ± 14.81*	64.51 ± 16.02*^,^^#^^,^^$^	<0.001
Creatinine (umol/L)	62.71 ± 14.18	70.29 ± 19.93	68.11 ± 22.80*	76.63 ± 30.27*^,^^$^	<0.001
FPG (mmol/L)	5.44 ± 0.85	5.54 ± 1.10	5.57 ± 1.01	5.40 ± 0.75	0.227
UA (umol/L)	298.08 ± 75.15	297.15 ± 77.20	324.66 ± 78.40*	331.79 ± 91.32*^,^^#^	<0.001
TC (mmol/L)	5.13 ± 1.11	4.89 ± 1.02	4.76 ± 1.14*	4.44 ± 1.06*^,^^#^^,^^$^	<0.001
HDL-C (mmol/L)	1.30 ± 0.34	1.30 ± 0.33	1.20 ± 0.30*	1.20 ± 0.27*	<0.001
LDL-C (mmol/L)	3.20 ± 0.90	2.99 ± 0.90	2.92 ± 0.89*	2.61 ± 0.84*^,^^#^^,^^$^	<0.001
TG (mmol/L)	1.78 ± 1.45	1.56 ± 0.93	1.70 ± 1.27	1.54 ± 0.84	0.253
Homocysteine (umol/L)	12.20 ± 6.11	14.73 ± 8.51	15.18 ± 8.38*	16.93 ± 7.83*	<0.001
RIMT (cm)	0.091 ± 0.023	0.099 ± 0.028	0.094 ± 0.023	0.102 ± 0.025*^,^^$^	0.038
LIMT (cm)	0.092 ± 0.023	0.114 ± 0.052	0.109 ± 0.083	0.110 ± 0.044	0.084

Note:

*vs group 1, p<0.05;

# vs group 2, p<0.05;

$ vs group 3, p<0.05. group 1: non-hypertension patients with CAVI<9; group 2: non-hypertension patients with CAVI ≧9; group 3: hypertension patients with CAVI<9; group 4: hypertension patients with CAVI ≧9. ACEI: angiotensin converting anzyme inhibitior; ARB: angiotensin Ⅱ receptor antagonist; CCB: calcium channel blocker; BMI: body mass index; CAVI: Cardio-ankle vascular index; SBP: systolic blood pressure; DBP: diastolic blood pressure; FPG: fasting plasma glucose; UA: uric acid; TC: cholesterol; LDL-C: low-density lipoprotein cholesterol; HDL-C: high-density lipoprotein cholesterol; TG: triglycerides; RIMT: right intima-media thickness; LIMT:left intima-media thickness.

**Fig 1:**
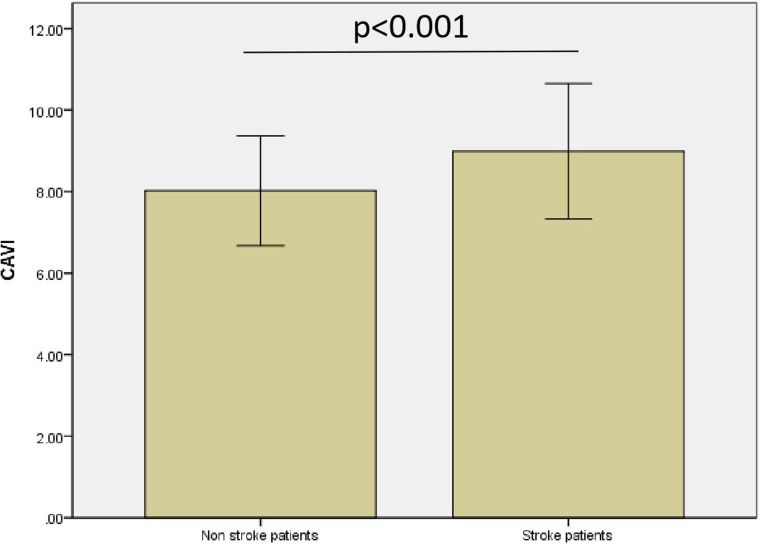
CAVI value in stroke patients and non-stroke patients.

In addition, the present study showed that level of CAVI was significantly higher in stroke patients than in non-stroke patients (8.99±1.66 vs 8.03±1.35, p<0.001), as shown in Fig 1.

### Pearson correlations between CAVI and metabolic markers

Next, we investigated the pearson correlations between CAVI and metabolic markers and other variables in the entire group. As shown in Table 3, our results showed that CAVI was positively correlated with age, SBP, pulse pressure, creatinine, and RIMT in entire study group. Negative correlation between CAVI and BMI was found in the entire group (r= -0.115, p<0.001).

**Table 3:** Pearman correlations between CAVI and study variables.

	All patients		Non-hypertension patients		Hypertension patients	
r	P value	r	P value	r	P value
Age (year)	0.485	<0.001	0.572	<0.001	0.402	<0.001
BMI(Kg/M^2)	−0.223	<0.001	−0.270	<0.001	−0.253	<0.001
SBP (mmHg)	0.192	<0.001	0.130	0.027	0.141	0.003
DBP (mmHg)	0.022	0.550	−0.050	0.396	−0.018	0.710
Pulse pressure (mmHg)	0.237	<0.001	0.226	<0.001	0.185	<0.001
Creatinine(umol/L)	0.131	<0.001	0.222	<0.001	0.068	0.153
FPG (mmol/L)	−0.035	0.338	0.099	0.093	−0.125	0.009
UA(umol/L)	−0.007	0.852	0.006	0.923	−0.064	0.185
TC (mmol/L)	−0.114	0.002	−0.019	0.752	−0.124	0.010
HDL-C (mmol/L)	−0.021	0.582	0.004	0.948	0.012	0.801
LDL-C (mmol/L)	−0.131	<0.001	−0.018	0.766	−0.156	0.421
TG (mmol/L)	−0.037	0.324	0.042	0.475	−0.086	0.074
Homocysteine (umol/L)	0.110	0.004	0.150	0.012	0.040	0.421
RIMT(cm)	0.113	0.044	0.122	0.166	0.089	0.226
LIMT(cm)	−0.053	0.346	0.264	0.002	−0.177	0.014

Note: BMI: body mass index; CAVI: Cardio-ankle vascular index; SBP: systolic blood pressure; DBP: diastolic blood pressure; FPG: fasting plasma glucose; UA: uric acid; TC: cholesterol; LDL-C: low-density lipoprotein cholesterol; HDL-C: high-density lipoprotein cholesterol; TG: triglycerides; RIMT: right intima-media thickness; LIMT:left intima-media thickness.

### Multiple linear regression analysis

Multiple linear regressions were used to estimate the coefficients of the linear equation, involving independent variables including age, gender, smoking, BMI, SBP, DBP, creatinine, FPG, uric acid, TC, TG, HDL-C, LDL-C, homocysteine, RIMT, LIMT and CAVI that affected the prevalence of stroke. As shown in Table 4, our results showed that CAVI and age were independent associating factors of stroke in all patients (β=0.268, p=0.040; β=0.135, p<0.001; respectively). And CAVI, DBP were independent associating factors of stroke in non-hypertension patients (Table 5, β=0.206, p=0.028; β=-0.192, p=0.040; respectively). CAVI was an independent associating factors of stroke in hypertension patients (Table 6, β=0.398, p<0.001).

**Table 4:** Multiple linear regression analysis for the relationship between stroke and study variables among entire study group.

	Unstandardized β	95% CI for β	Std. Error	Standardized β	t	P value
constant	−0.705	[−1.010, −0.041]	0.155	–	−4.562	<0.001
CAVI	0.081	[0.040, 0.122]	0.021	0.268	3.911	0.040
Age (year)	0.005	[0.000, 0.009]	0.002	0.135	1.973	<0.001

Note: CAVI: Cardio-ankle vascular index.

**Table 5:** Multiple linear regression analysis for the relationship between stroke and study variables among non-hypertension group.

	Unstandardized β	95% CI for β	Std. Error	Standardized β	t	P value
constant	0.328	[−0.385, 1.040]	0.359	–	0.912	0.364
CAVI	0.057	[0.006, 0.107]	0.025	0.206	2.233	0.028
DBP	−0.007	[−0.014, 0.000]	0.003	−0.192	−2.079	0.040

Note: CAVI: Cardio-ankle vascular index; DBP: diastolic blood pressure

**Table 6:** Multiple linear regression analysis for the relationship between stroke and study variables among hypertension group.

	Unstandardized β	95% CI for β	Std. Error	Standardized β	t	P value
constant	−0.807	[−1.215, −0.399]	0.207	–	−3.907	<0.001
CAVI	0.130	[0.083, 0.178]	0.024	0.398	5.419	<0.001

Note: CAVI: Cardio-ankle vascular index.

In addition, further analysis showed that odds ratio of stroke was 2.722 in hypertension patients with CAVI ≧9 than in hypertension patients with CAVI<9 (95%CI: [1.964, 3.771]. Odds ratio of stroke was 2.489 in non-hypertension patients with CAVI ≧9 than in non-hypertension patients with CAVI<9 (95%CI: [1.387, 4.466].

## Discussion

The present study showed that the prevalence of stroke was higher in hypertension patients with CAVI ≧9 than in hypertension patients with CAVI<9, with higher level of right intima-media thickness. CAVI was an independent associating factors of stroke in hypertension patients.

The rapid economic transformations contributed to the ageing demography, unhealthy lifestyles, and environmental changes. Rising cerebrovascular diseases rates have had a major economic impact, which has challenged the healthcare system and the whole society [11]. Research has showed that the burden of stroke in China is increasing year by year, especially in rural areas, where stroke is associated with hypertension, smoking, and other factors [12]. Arteriosclerosis evaluated by arterial stiffness is the pathophysiological change of stroke. Arterial stiffness could be measured by CAVI. The characteristics of CAVI differ from other physiological tests of arterial stiffness due to the independency from blood pressure at the time of examination [13]. Our present study showed that CAVI was significantly higher in hypertension patients, the same to our previous research [14]. In addition, our present study showed that the level of homocysteine and prevalence of coronary artery disease was higher in hypertension patients, similar results to our previous study [15,16]. CAVI was a sensitive marker of the arterial aging process, above and beyond conventional upper arm blood pressure [17]. Recent studies have showed that the level of CAVI in patients with metabolic syndrome was significantly elevated, and the elevation of CAVI was associated with an increase in the components of metabolic syndrome that accompany the patient [18]. Patients with type 2 diabetes had an increased risk of arterial stiffness, based on the CAVI score, compared with nondiabetic patients [19]. Male and age were significantly associated with CAVI≥9 [20]. Increased CAVI was independently associated with chronic kidney disease in patients with diabetes mellitus [21]. Takenaka T suggested that CAVI can be used as a screening test to detect for the presence of cardiovascular diseases in patients undergoing hemodialysis [22]. Above studies showed that CAVI was a reliable evaluation indicator for vascular-related diseases [23].

Our present study showed that the prevalence of stroke was higher in hypertension patients with CAVI ≧9 than in hypertension patients with CAVI<9, that was to say, the arterial stiffness evaluation was very important in hypertension patients. In addition to the therapy of hypertension, some parameters such as SBP, DBP and pulse pressure should be considered, the arterial stiffness should also be fixed in the process of follow up. And our present study showed that RIMT was higher in hypertension patients with CAVI ≧9 than in hypertension patients with CAVI<9. We thought that with the stiffening of artery, the IMT would become thicker and carotid plaque could be generated. And plaque stability become worse during the stiffening of artery. Carotid IMT was independently associated with stroke among hypertensive patients [24]. And previous study showed that carotid IMT was associated with arterial stiffness in patients with diabetes mellitus and without diabetes mellitus [25]. And previous study showed that CAVI was correlated with IMT and much stronger correlated with the plaque score [26]. CAVI was higher in patients with cerebral infarction [27] and CAVI reflected cerebral small-vessel diseases in healthy young and middle-aged individuals [28]. Arterial stiffness was associated with decreased cognitive function [29]. Above studies showed that carotid atherosclerosis and arterial stiffness were both related to risk factors associated with the occurrence of stroke.

As we all know, cardiovascular and cerebrovascular diseases have become an important factor threatening human health all over the world. The pathophysiological changes of cardiovascular and cerebrovascular diseases are local vascular function or structural abnormalities, manifested as endothelial dysfunction, arteriosclerosis, atherosclerosis, etc., which lead to lumen stenosis or acute cardiovascular and cerebrovascular events. Therefore, if we can assess vascular dysfunction early, detect abnormalities in time and give early intervention, it is possible to reverse abnormal vascular functions, thereby reducing the incidence of cardiovascular and cerebrovascular events, which is of great significance for the prevention and treatment of cardiovascular and cerebrovascular diseases. Our research and previous studies have showed that CAVI, as a non-invasive indicator for detecting vascular function, has the characteristics of simplicity, operability, and repeatability. It is suitable for use in medical institutions at all levels, especially in primary hospitals, and is suitable for the management of chronic cardiovascular and cerebrovascular diseases [30].

A major limitation of our study is its cross-sectional design; another limitation is that the patients’ numbers of each group including gender composition were not balanced. And we did not include the physical activity in this study, which might affect the value of CAVI. This might caused some bias, so large sample and prospective study need to be investigated in future.

## Conclusions

Prevalence of stroke was higher in hypertension patients with CAVI ≧9 than in hypertension patients with CAVI<9, with higher level of right intima-media thickness. CAVI was an independent associating factors of stroke in hypertension patients.
